# An allosteric DNAzyme-Based modular system for dynamic computation and sensitive MicroRNA detection

**DOI:** 10.3389/fbioe.2026.1895332

**Published:** 2026-07-29

**Authors:** Pali Ye, Sirui Li, Zhen Xiong, Mianfeng Zeng, Yuhai Li, Xin Li, Xiaolong Shi

**Affiliations:** 1 Institute of Computing Science and Technology, Guangzhou University, Guangzhou, China; 2 Department of Natural Sciences, Manchester Metropolitan University, Manchester, United Kingdom; 3 Department of Gynecology, Renmin Hospital of Wuhan University, Wuhan, China

**Keywords:** allosteric DNAzyme, DNA strand displacement, entropy-driven catalysis, fluorescence biosensor, molecular computation

## Abstract

**Introduction:**

Circulating microRNAs (miRNAs) are widely studied as biomarkers for early diagnosis and disease follow-up, but their analysis is still limited by low abundance, short length, and high sequence similarity among miRNA family members. DNAzymes and entropy-driven catalysis (EDC) provide useful tools for nucleic acid sensing without exogenous protein enzymes. However, in many existing designs, signal amplification and molecular computation are treated as separate processes, and the amplified target signal is not readily converted into inputs for downstream logic circuits. To address this issue, we designed an allosterically regulated DNAzyme platform in which miRNA sensing and molecular computation are constructed from compatible nucleic acid modules.

**Methods:**

In the computing unit, controlled activation of DNAzymes was used to implement co-activated AND logic, thresholding, and subtraction. These gates were then connected through orthogonal sequence domains to form an integrated logic circuit capable of cascaded signal processing. In the sensing unit, an EDC reaction was coupled with DNAzyme-mediated cleavage to establish a self-feedback amplification pathway. First, the target miRNA initiates a strand displacement reaction to release an active DNAzyme. Subsequently, this DNAzyme cleaves its substrate to generate an initial fluorescence output and simultaneously release a secondary trigger for downstream signal amplification.

**Results:**

Using miRNA-10b as a model target, the biosensor operated under isothermal conditions without exogenous protein enzymes and showed a linear response from 50 pM to 5 nM, with a detection limit of 30 pM. The assay also distinguished miRNA-10b from single-base mismatched sequences and non-target miRNAs.

**Discussion:**

This work links EDC-based amplification with allosteric DNAzyme computation, offering a programmable strategy for nucleic acid systems that combine biomarker recognition with molecular information processing.

## 1 Introduction

MicroRNAs (miRNAs) are short non-coding RNAs that regulate gene expression and participate in diverse physiological and pathological processes. Aberrant miRNA expression has been associated with cancers, cardiovascular diseases, inflammatory disorders, and metabolic dysfunctions, making circulating miRNAs attractive biomarkers for early diagnosis, prognosis evaluation, and disease monitoring (Condrat et al., 2020; Au Yeung et al., 2016; Asjad and Dobrzynski, 2023; Lu et al., 2005). However, accurate miRNA analysis remains technically challenging because miRNAs are usually short, low-abundance, highly homologous among family members, and susceptible to interference from complex biological matrices (Pan et al., 2020; Pritchard et al., 2012). Conventional analytical methods, including quantitative reverse transcription polymerase chain reaction, *in situ* hybridization, and microarray analysis, have greatly advanced miRNA profiling (Kim et al., 2018; Ban and Song, 2022; Urbanek et al., 2015; Song et al., 2010; Castoldi et al., 2006; Baskerville and Bartel, 2005). Nevertheless, these methods often require enzymatic conversion, thermal cycling, sophisticated instrumentation, or complex sample preparation, which limits their direct integration into simple, programmable, and isothermal molecular diagnostic systems.

Programmable nucleic acid reaction networks provide an attractive route for addressing these limitations. DNA strand displacement is a fundamental mechanism in dynamic DNA nanotechnology, enabling sequence-programmed molecular reactions through toehold-mediated hybridization and branch migration.

Based on this principle, entropy-driven catalysis (EDC) has emerged as a representative amplification strategy that does not require exogenous protein enzymes. In an EDC system, a target strand catalytically triggers the reconfiguration of metastable DNA complexes, while a fuel strand drives the reaction forward by increasing the entropy of the system, thereby converting a weak nucleic acid input into amplified molecular outputs under isothermal conditions (Finn et al., 2007; Wang et al., 2024). Recent EDC-based systems have been coupled with colorimetric, fluorescent, electrochemical, and DNAzyme-assisted readout strategies for detecting nucleic acids and sequence variants, demonstrating the modularity and analytical potential of EDC in biosensing (Zhao et al., 2023; Zhang et al., 2025). DNAzymes offer another important class of programmable nucleic acid tools. As catalytic DNA molecules, DNAzymes combine the sequence programmability of DNA with enzyme-like catalytic functions (Shi et al., 2024). RNA-cleaving DNAzymes are particularly useful because they can catalyze site-specific substrate cleavage and generate measurable fluorescence, colorimetric, or electrochemical signals (Zhang et al., 2024). DNAzyme-mediated assays have been widely applied for amplified detection of nucleic acids, pathogens, metal ions, and other biomarkers (Wang et al., 2017; Dong et al., 2024; Daus et al., 2022; Ding et al., 2023; Liu et al., 2025a; Liu et al., 2025b). Compared with protein enzymes, DNAzymes are easier to synthesize, more chemically stable, and highly compatible with strand-displacement circuits. Importantly, DNAzyme activity can be controlled by inhibitor strands, target-induced conformational changes, or allosteric regulatory domains. Therefore, DNAzymes can serve not only as signal-generating elements for biosensing, but also as programmable catalytic units for molecular information processing.

Beyond biosensing, EDC, DNAzymes, and strand-displacement reactions have also contributed to the development of molecular computation. DNA-based molecular machines and nanodevices have shown that nucleic acid structures can perform dynamic and programmable functions (Şaş et al., 2025; Peng et al., 2018). Enzyme-operated DNA nanodevices and biocatalytic logic gates have demonstrated molecular-level signal processing (Kirichenko et al., 2023). DNAzymes have been used to construct autonomous walkers, computing circuits, multiplexers, demultiplexers, and logic-gate-mediated self-assembly systems (Liu et al., 2024; Zhang et al., 2026; Abendroth et al., 2015; Del Grosso et al., 2015). Meanwhile, strand-displacement-based circuits have enabled arithmetic operations, timer circuits, and increasingly complex DNA computing architectures (Fratto and Katz, 2016; Breaker and Joyce, 1995). More recently, logic-guided DNA framework systems have suggested the possibility of integrating molecular computation with precision diagnostics (Tian et al., 2005). These studies indicate that nucleic acid systems are no longer limited to passive target recognition, but can be programmed to execute signal processing, logic computation, and hierarchical molecular operations.

Although DNA walkers, electrochemical cascade amplification, and enzyme-assisted strand displacement have improved the sensitivity of miRNA assays, most of these systems are mainly designed for final signal readout. Recently, several highly sensitive miRNA detection strategies have been reported, including spatial confinement-based nucleic acid amplifiers (Xia et al., 2025b), CRISPR/Cas12a-coupled strand displacement (Feng et al., 2023), enzyme-activated DNA sensors (Xia et al., 2025a), and optically controlled cascade reactions (Zhao et al., 2021). Despite their excellent analytical performance, the amplified products in many sensing systems usually terminate as fluorescence, electrochemical, or imaging outputs and are not readily converted into standardized molecular inputs for downstream logic circuits. EDC-based biosensors are attractive because of their modular nature and because they do not require exogenous protein enzymes, yet many EDC systems mainly function as amplification modules and do not provide logic-compatible outputs for higher-order computing. DNAzyme-based logic circuits, including DNAzyme computing circuits, multiplexers, demultiplexers, and logic-gate-mediated DNA self-assembly, have demonstrated the programmability of catalytic nucleic acid circuits (Elbaz et al., 2010; Orbach et al., 2014; Zhang et al., 2016; Zhang and Seelig, 2011). However, direct use of DNAzyme cleavage products as inputs for downstream logic gates may suffer from signal attenuation and reduced reaction efficiency. As a result, many reported systems are still dominated by relatively simple Boolean operations or static signal conversion. More advanced operations, such as threshold activation, subtraction, and multi-layer signal integration, remain underdeveloped in DNAzyme-based systems. In addition, molecular logic networks usually require input signals above a certain activation threshold, whereas clinically relevant miRNAs are often present at trace levels. Therefore, a key challenge is to construct a unified molecular platform that can integrate signal amplification with logic-compatible molecular signal processing. Herein, we developed an allosteric DNAzyme-based modular system that integrates EDC amplification, DNAzyme self-feedback, and molecular logic computation within a single nucleic acid framework. In the present design, the computing and sensing parts are built within the same DNAzyme-centered reaction framework. For the computing part, allosteric control of DNAzyme activity was used to realize three operations: co-activated AND logic, threshold response, and subtraction. These gates were linked through mutually independent sequence domains, allowing the product of one reaction to serve as the input for the next and forming a programmable DNAzyme cascade for stepwise signal processing. For the sensing part, miRNA recognition was coupled to an EDC-DNAzyme amplification route via three sequential steps: (1) Target Recognition, where the target miRNA initiates the EDC reaction and releases an active DNAzyme; (2) Signal Transduction, where this DNAzyme cleaves a hairpin substrate to produce a fluorescence-generating strand; and (3) Signal Amplification, where a simultaneously released secondary trigger re-enters the EDC cycle. In this way, target recycling and DNAzyme-mediated feedback are clearly and efficiently combined in one amplification process. MiRNA-10 b was used as a model target to examine the sensing performance. Overall, the proposed design connects EDC-based amplification with allosteric DNAzyme logic gates, providing a nucleic acid reaction scheme in which low-level biomarker signals can be amplified before being processed by molecular computation.

## 2 Materials and methods

### 2.1 Materials

All oligonucleotides were obtained from Sangon Biotech Co., Ltd. (Shanghai, China). These included DNAzyme strands, inhibitory strands, fuel strands, substrates, reporter probes, miRNA targets, and mismatched control sequences. Strands without chemical modification were purified by PAGE, while oligonucleotides containing ribonucleotide sites, fluorophores, or quenchers were purified by HPLC. The dried oligonucleotides were reconstituted in ultrapure water and their concentrations were determined from the absorbance at 260 nm using a NanoDrop spectrophotometer. The prepared stock solutions were stored at 4 °C before use, and fluorescently labeled strands were kept away from light during preparation and storage. Unless otherwise noted, analytical-grade reagents were used directly without additional purification. Detailed sequence information and chemical modifications are provided in Supplementary Tables S2, S3.

### 2.2 Preparation of DNA samples and logic gates

The DNA logic gates were prepared according to their structural requirements using either a two-step annealing procedure or a single-step incubation procedure. For the first type of logic gate, in which the catalytic activity of the DNAzyme needed to be temporarily inhibited before substrate binding, a two-step annealing process was performed. In the first step, the DNAzyme strand and the corresponding short inhibitor strand were mixed in 1 × TAE buffer containing 12.5 mM $Mg^{2+}$ at pH 8.0. The mixture was heated to 95 °C for 4 min to disrupt potential secondary structures and was then slowly cooled to 20 °C at a rate of 0.5 °C/min using a PCR thermocycler, allowing the DNAzyme–inhibitor complex to form. In the second step, the substrate strand was added to the pre-annealed DNAzyme–inhibitor complex. The final reaction buffer was maintained at 1 × TAE containing 12.5 mM $Mg^{2+}$ at pH 8.0, and the mixture was incubated at 20 °C for 4 h to promote complete assembly of the allosterically regulated DNAzyme logic gate. For the second type of logic gate, which did not require pre-hybridization between the DNAzyme and an inhibitor strand, the required DNA components were directly mixed in the same reaction buffer and incubated at 20 °C for 4 h. The assembled DNA complexes were then used for subsequent PAGE characterization and fluorescence kinetic analysis.

### 2.3 Polyacrylamide gel electrophoresis (PAGE) analysis

After preparation, DNA samples were mixed with 6 × loading buffer and loaded onto a 12% native polyacrylamide gel. Electrophoresis was carried out at a constant voltage of 100 V for approximately 90 min at room temperature, until the bromophenol blue tracking dye migrated close to the bottom of the gel. The gel was then stained with GelRed nucleic acid dye for 30 min. Finally, the DNA bands were visualized and recorded using an ultraviolet gel imaging system.

### 2.4 Real-time fluorescence kinetic analysis

Fluorescence monitoring and kinetic analyses were performed using the plate-reading module of a QuantStudio 3 Real-Time PCR System equipped with a 96-well plate format. Unless stated otherwise, fluorescence measurements were performed at 25 °C. ROX, TET, and FAM were used as the fluorescent reporters in different reaction systems. The excitation/emission settings were 588/608 nm for ROX, 521/536 nm for TET, and 494/518 nm for FAM. During data analysis, the blank reaction was taken as the reference background. The fluorescence response of each sample was expressed as Rn, defined as the ratio between the sample signal and the blank signal (Rn = $F_{\mathrm{sample}}/F_{\mathrm{blank}}$ ). Immediately after the reaction components were mixed, the samples were placed into the qPCR instrument for measurement. This procedure helped capture the early stage of the reaction and reduced the loss of kinetic information caused by premature signal generation before detection.

## 3 Results

### 3.1 Construction and validation of the DNAzyme-based integrated logic circuit

We first built a molecular computing unit based on allosterically regulated DNAzyme gates. The three gates were designed to carry out AND logic operations, threshold response, and subtraction, which together act as a versatile information processing unit. In this design, the co-activated AND gate performs a logical conjunction operation, the threshold gate provides a binary ON/OFF switching step, and the subtraction gate processes the difference between positive and negative signals. These gates were then connected through independent sequence domains, allowing the output of one module to trigger the next reaction and form a cascaded DNAzyme logic network.

**FIGURE 1:**
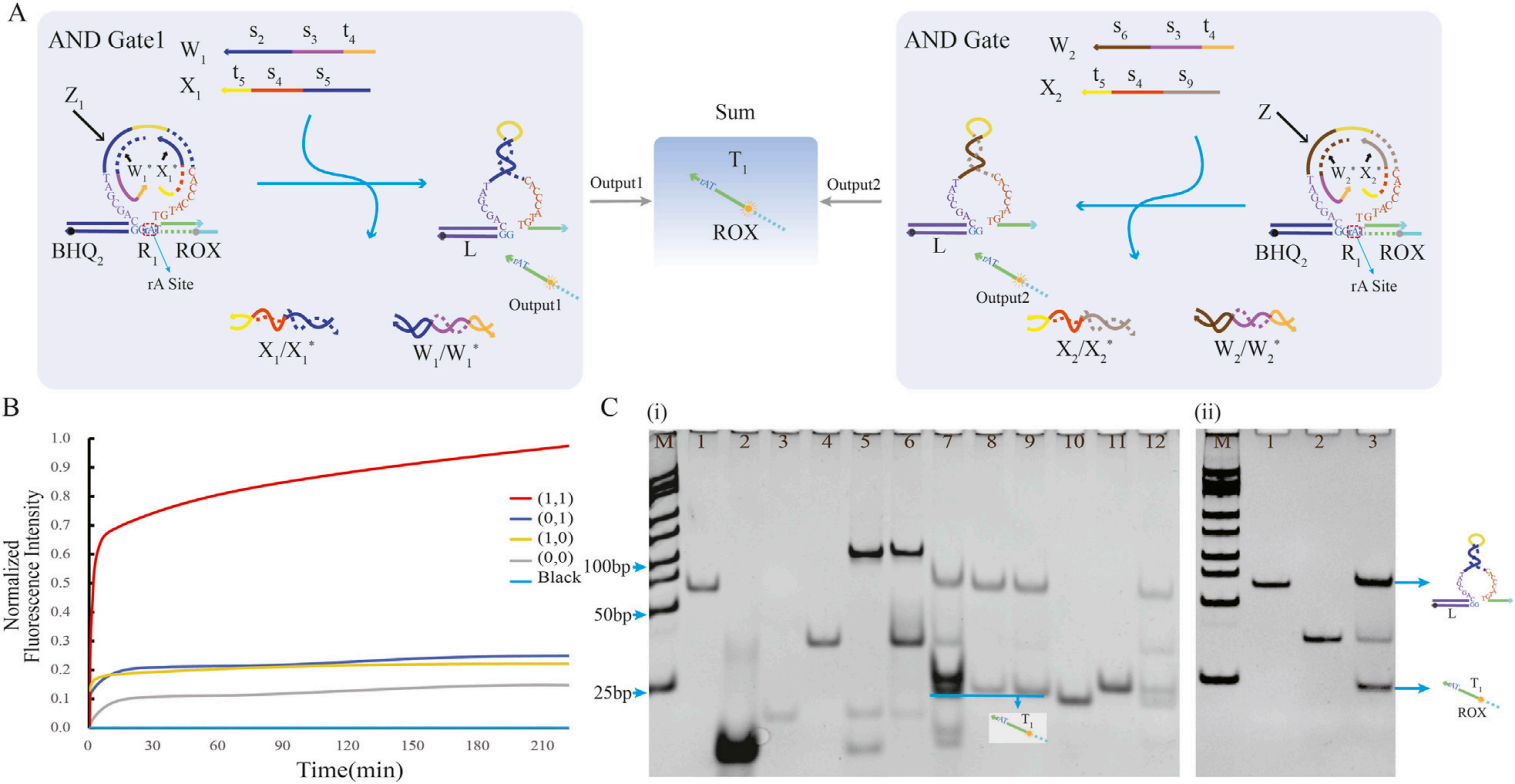
Design and verification of the co-activated AND gate. **(A)** Schematic illustration of the co-activated AND module. **(B)** Real-time fluorescence quantitative PCR kinetics. The curves represent different input conditions: W1 + X1 + Gate1 (red), W1 + Gate1 (dark blue), X1 + Gate1 (yellow), isolated Gate1 (Z1-W1*-X1*-R1) complex (gray), and the blank control (light blue). **(C)** PAGE analysis. (i) Verification of complex assembly: Lane M is the marker; Lanes 1–4 are single strands Z1, W1*, X1*, and R1, respectively; Lane 5 is the Z1-W1*-X1* complex; Lane 6 is the fully assembled Gate1 (Z1-W1*-X1*-R1) complex; Lanes 7 and 12 are the reaction products of the Gate1 complex triggered by W1 and X1; Lanes 8 and 9 are the Z1-R1 complex; Lanes 10 and 11 are single strands W1 and X1, respectively. (ii) Verification of catalytic cleavage: Lanes 1 and 2 are single strands Z1 and R1, respectively; Lane 3 is the Z1-R1 cleavage reaction complex. Note: The apparent migration of single-stranded cleavage products should not be directly compared with double-stranded DNA markers. Under native PAGE conditions, migration is affected not only by nucleotide length, but also by secondary structure, strand flexibility, base composition, and chemical modifications.

The co-activated AND gate was first designed to convert paired input signals into a catalytic fluorescence output (Figure 1A). In the logic gate, the allosteric regulatory strands W1* and X1* act as programmable regulators that define the helper and input recognition domains, respectively. Specific hybridization between W1/W1* and X1/X1* induces strand displacement and releases the inhibition imposed on the DNAzyme. In this design, the catalytic activity of Z1 is restored only when the corresponding input and helper strands are simultaneously present, thereby achieving an AND logic operation at the molecular level. Once activated, Z1 cleaves R1 at the embedded rA site, separates the fluorophore from the quencher, and generates a fluorescence signal that can be monitored in real time. At the molecular level, this cleavage is driven by a metal-ion-dependent transesterification reaction. Specifically, $Mg^{2+}$ ions within the buffer facilitate the deprotonation of the 2′-hydroxyl (2′-OH) group on the rA residue, triggering a nucleophilic attack on the adjacent phosphodiester bond and leading to strand cleavage.

**FIGURE 2:**
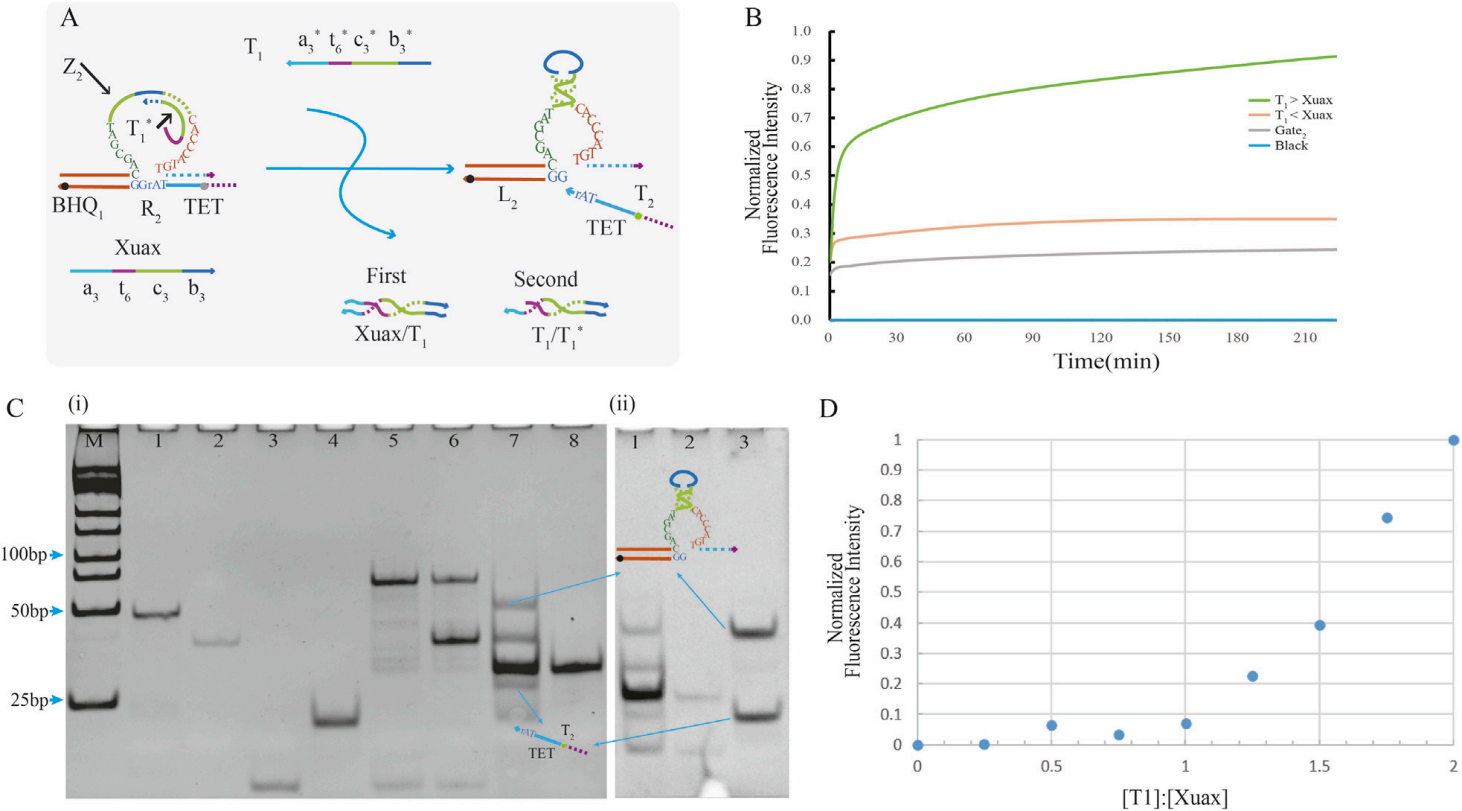
Design and verification of the threshold gate. **(A)** Schematic illustration of the molecular design of the threshold gate. **(B)** Real-time fluorescence quantitative PCR kinetics. The green and light orange curves represent the reaction systems of Gate2 triggered when [T1]>[Xuax] and [T1]<[Xuax] , respectively. The gray curve represents the isolated Gate2 (Z2-T1*-R2) complex, and the light blue curve is the blank control. **(C)** Native polyacrylamide gel electrophoresis (PAGE) analysis. (i) Verification of complex assembly: Lane M is the marker; Lanes 1–4 are single strands Z2, R2, T1*, and Xuax, respectively; Lane 5 is the Z2-T1* complex; Lane 6 is the Gate2 (Z2-T1*-R2) complex; Lane 7 is the reaction product of T1 interacting with the Z2-T1*-R2-Xuax system; Lane 8 is the T1-Xuax complex. (ii) Verification of catalytic cleavage: Lane 1 is the reaction product of T1 interacting with the Z2-T1*-R2-Xuax system; Lane 2 is single strand T2; Lane 3 is the Z2-R2 cleavage reaction complex. Note: The apparent migration of single-stranded cleavage products should not be directly compared with double-stranded DNA markers. Under native PAGE conditions, migration is affected not only by nucleotide length, but also by secondary structure, strand flexibility, base composition, and chemical modifications. **(D)** Normalized threshold switching curve of Gate2 as a function of the [T1]/[Xuax] ratio. The concentration of Xuax was kept constant, while the concentration of T1 was varied to obtain T1:Xuax ratios of 0.25:1, 0.5:1, 0.75:1, 1:1, 1.25:1, 1.5:1, 1.75:1, and 2:1. The normalized fluorescence response was calculated from the endpoint fluorescence intensity using $\left(F-F_{\mathrm{blank}}\right)/\left(F_{\max}-F_{\mathrm{blank}}\right)$ . Note: The apparent migration of single-stranded cleavage products should not be directly compared with double-stranded DNA markers. Under native PAGE conditions, migration is affected not only by nucleotide length, but also by secondary structure, strand flexibility, base composition, and chemical modifications.

The co-activated AND gate was validated by native PAGE and fluorescence kinetic analysis. As shown in Figure 1Cii, Z1 specifically cleaved the substrate R1, producing the expected cleavage products and confirming its catalytic activity. Figure 1Ci further verified the assembly and activation of Gate1. The slowest-migrating band in lane 6 corresponds to the fully assembled Gate1 complex. After simultaneous addition of W1 and X1, a new product band appeared, indicating input-triggered activation of the DNAzyme gate and release of the downstream output strand. Consistent with the gel results, fluorescence kinetic analysis showed that a strong fluorescence increase was observed only in the presence of both W1 and X1, whereas the no-input and single-input controls remained at low background levels (Figure 1B). These results demonstrate that the AND gate can achieve logic operations and convert the recognition event into a measurable catalytic output. Next, we constructed a threshold gate to introduce a thresholding function into the molecular computing system (Figure 2A). The threshold gate regulates the downstream activation of DNAzyme Z2 through a threshold strand, Xuax. When the concentration of input T1 is lower than or comparable to that of Xuax, T1 is preferentially sequestered by Xuax and cannot efficiently activate the Z2-based DNAzyme complex. In contrast, when T1 exceeds the threshold concentration, the excess T1 displaces the inhibitor strand T1* from the Z2-T1*-R2 complex, restoring the catalytic activity of Z2. Activated Z2 then cleaves substrate R2 and generates the downstream output T2 together with a fluorescence signal. This design allows the gate to maintain a low-output state below the threshold and generate a high-output response once the input exceeds the preset threshold.

The threshold behavior of Gate2 was experimentally examined by PAGE analysis, fluorescence kinetic analysis, and an additional input-to-threshold ratio experiment. In the PAGE assay, when the T1 concentration exceeded that of Xuax, a distinct T1-Xuax waste band and a new product band were observed (Figure 2C–I). The mobility of this product was consistent with the cleavage product generated by the Z2-R2 control system (Figure 2C–2ii), indicating activation of the DNAzyme. Fluorescence kinetic analysis further supported threshold-dependent ON/OFF behavior under the low-input and high-input conditions tested (Figure 2B). To further characterize the switching behavior around the threshold point, we measured the normalized fluorescence response under a series of [T1]/[Xuax] ratios while keeping the concentration of Xuax constant. The T1:Xuax ratios were set as 0.25:1, 0.5:1, 0.75:1, 1:1, 1.25:1, 1.5:1, 1.75:1, and 2:1. When the [T1]/[Xuax] ratio was below 1.0, the normalized fluorescence response remained relatively low, indicating that most T1 was sequestered by the threshold strand Xuax. As the ratio approached and exceeded 1.0, the fluorescence response increased, showing that excess T1 was able to activate the Z2-T1*-R2 complex. The resulting normalized endpoint fluorescence response exhibited a ratio-dependent switching profile, further confirming the threshold-controlled activation behavior of Gate2 (Figure 2D).

**FIGURE 3:**
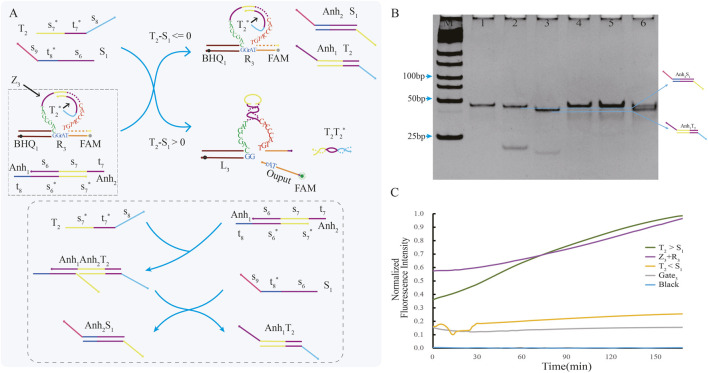
Design and verification of the subtraction gate. **(A)** Schematic illustration of the molecular design of the subtraction gate. **(B)** Native polyacrylamide gel electrophoresis (PAGE) analysis: Lane M is the marker; Lanes 1–3 are the Anh1-Anh2, Anh1-T2, and Anh2-S1 complexes, respectively; Lanes 4 and 5 are the reaction products of the single input strand (T2 or S1) interacting with the Anh1-Anh2 complex, respectively; Lane 6 is the reaction product of both input strands (T2 and S1 simultaneously) interacting with the Anh1-Anh2 complex (the concentration ratio of all reaction components is 1:1). **(C)** Real-time fluorescence quantitative PCR kinetics. The purple and yellow curves represent the reaction systems of Gate3 triggered under the conditions of T2>S1 and T2<S1 , respectively. The dark green curve represents the free Z3+R3 (full cleavage) positive control system, the gray curve is the isolated Gate3 (Z3−T2∗−R3) complex, and the light blue curve is the blank control.

We then designed a subtraction gate to enable differential signal processing (Figure 3A). This gate is based on a molecular annihilation strategy, in which the double-stranded Anh1/Anh2 complex serves as a competitive middleware to balance the positive input T2 and the negative input S1. When T2 and S1 are both present, they are consumed through competitive hybridization with the annihilation middleware. Free T2 remains only when the concentration of T2 is higher than that of S1. The remaining T2 can then remove the inhibitor strand T2* from the Z3-T2*-R3 complex, thereby activating Z3. Activated Z3 cleaves substrate R3 and generates the final fluorescence output. Thus, the subtraction gate produces an output only when the positive signal exceeds the negative signal.

Native PAGE confirmed the formation and operation of the annihilation middleware (Figure 3B). Single-input reactions with either T2 or S1 did not produce the same annihilation product as the dual-input reaction. In contrast, when both T2 and S1 were present, a stable annihilation complex was formed, confirming the intended competitive interaction. Fluorescence kinetic analysis further supported the subtraction logic (Figure 3C). When the positive input T2 exceeded the negative input S1, residual T2 activated Gate3 and produced a strong fluorescence response. When T2 was insufficient, most T2 was consumed by the annihilation reaction, and the fluorescence signal remained low. These results verify that the subtraction gate can transform the concentration difference between positive and negative inputs into a catalytic fluorescence output.

**FIGURE 4:**
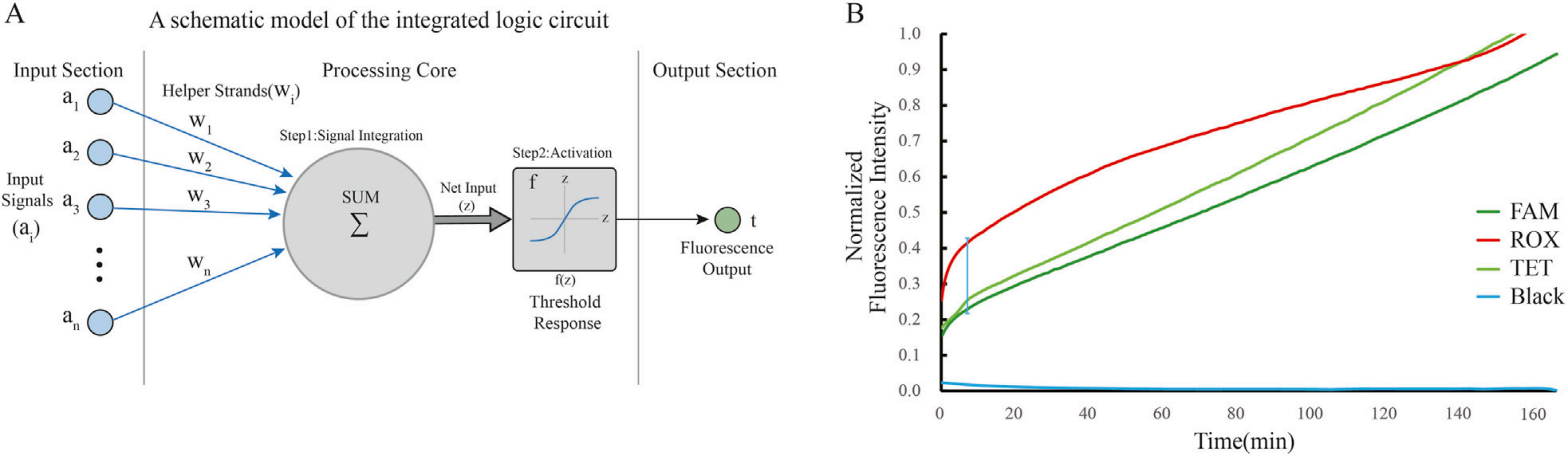
Construction and validation of the integrated logic circuit. **(A)** Schematic illustration of the cascaded computational model. **(B)** Multi-channel real-time fluorescence quantitative PCR kinetics of the cascade network. The concentration ratio of the reaction components in the system is [W1]: [X1] [Gate1]: [Gate2] [Gate3] = 1.5:1.5:1.2:1.2:1, with [W1] = [X1] = 1.0 μ M, [Gate1] = [Gate2] = 0.8 μ M, and [Gate3] = 0.67 μ M.

The cascade reaction was followed in three fluorescence channels, corresponding to ROX, TET, and FAM (Figure 4B). After W1 and X1 were introduced, the signal from Gate1 increased first, showing that the AND logic integration module had been triggered and that T1 was released for the next step. T1 then entered the threshold module. Once its level was sufficient to overcome the Xuax-defined threshold, Gate2 was activated and produced T2, accompanied by a second fluorescence increase. The generated T2 further triggered Gate3, where the subtraction module converted the upstream signal into the final FAM output. The sequential increase in fluorescence across the three channels confirms successful cross-layer signal transmission from AND logic integration to threshold activation and final subtraction. Together, these results demonstrate that allosteric DNAzyme gates can be modularly integrated into an integrated molecular computing network capable of hierarchical signal processing tailored for practical diagnostic applications.

### 3.2 Development and validation of an EDC-DNAzyme cascaded amplification biosensor for miRNA detection

Although the DNAzyme-based integrated logic network demonstrated programmable signal integration and thresholding, its practical use in biomarker analysis requires a sensing interface capable of converting trace nucleic acid targets into robust molecular signals. Circulating miRNAs are typically present at low concentrations and may be insufficient to directly activate downstream molecular logic circuits. Therefore, we designed a cascaded fluorescence biosensor that operates without exogenous protein enzymes by integrating entropy-driven catalysis (EDC) with DNAzyme-mediated self-feedback amplification. This sensing front-end was intended to amplify weak miRNA inputs and generate fluorescence outputs that are compatible with downstream logic analysis for reliable diagnostics.

**FIGURE 5:**
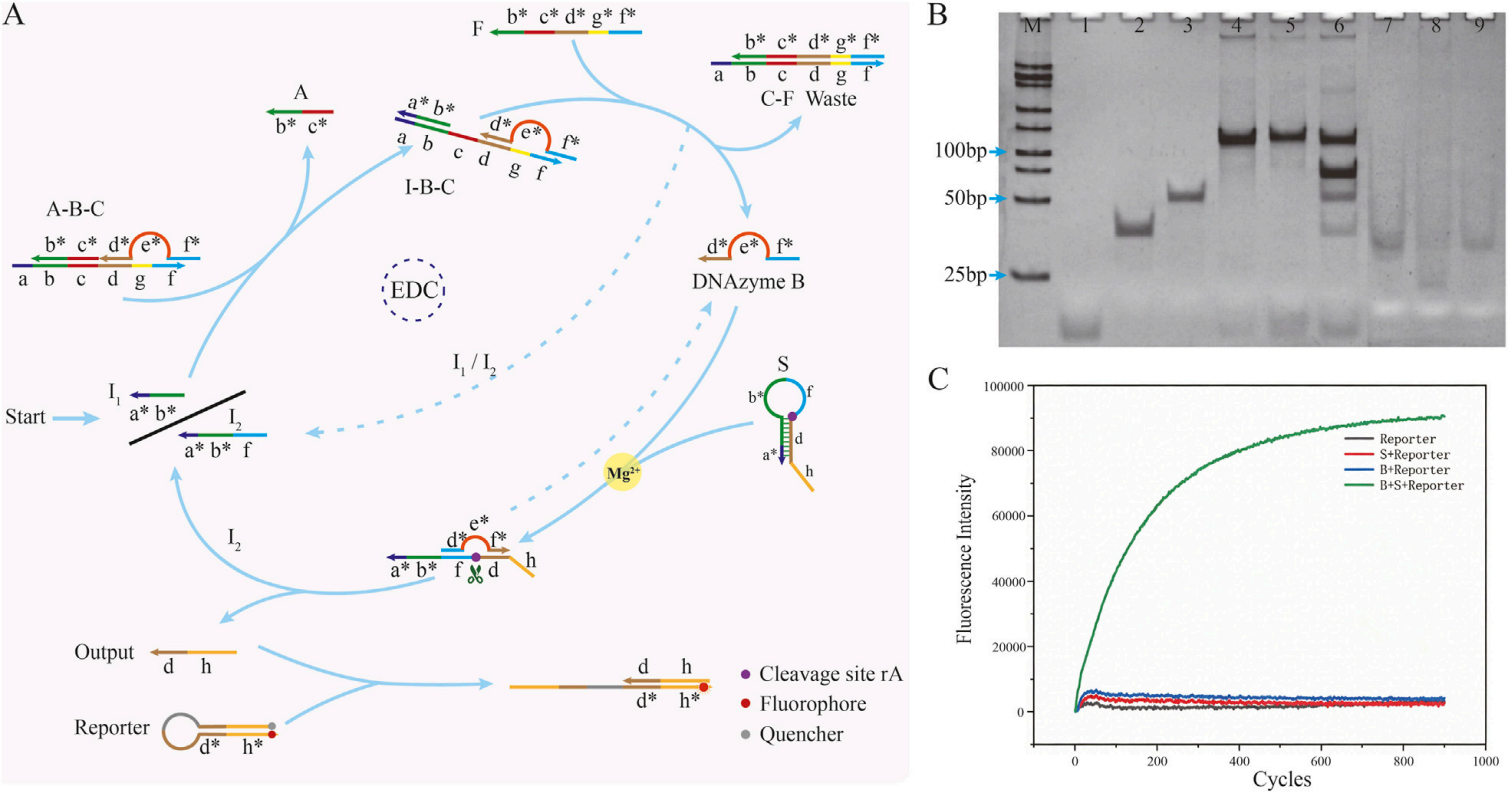
Design and verification of the entropy-driven catalysis-based self-feedback amplification biosensor. **(A)** Schematic illustration of the self-feedback amplification biosensor. **(B)** PAGE analysis: Lane M is the marker; Lanes 1–4 are single strand A, free DNAzyme (single strand B), fuel strand F, and the initial A-B-C complex, respectively; Lane 5 is the reaction product of target I1 interacting with the A-B-C complex; Lane 6 is the reaction product of both target I1 and fuel strand F interacting with the A-B-C system; Lanes 7–9 are the mixture of strand F and hairpin substrate S, the catalytic cleavage product of strand B and substrate S, and the isolated single strand S, respectively. **(C)** Fluorescence emission spectra of different reaction systems: (1) Reporter complex only; (2) Substrate S + Reporter; (3) Free strand B+ Reporter; (4) Substrate S + strand B+ Reporter (representing the complete catalytic output signal).

The sensing mechanism consists of two coupled stages: an EDC cycle and a DNAzyme self-feedback amplification loop (Figure 5A). The sensing reaction begins when I1 binds to the A-B-C complex and initiates strand displacement. With the assistance of fuel strand F, I1 is released for another reaction cycle, while strand B is liberated as an active DNAzyme. The released B strand then acts on the hairpin substrate S and cleaves it at the ribonucleotide-containing site. This cleavage produces two functional products: one strand activates the reporter probe to generate fluorescence, and the other, I2, serves as a new trigger for the A-B-C complex. As I2 feeds back into the upstream EDC reaction, the initial target-recognition event is converted into a repeated amplification process. In this way, target recycling and DNAzyme-mediated feedback work together to strengthen the fluorescence signal under isothermal conditions without exogenous protein enzymes.

Native PAGE was used to examine whether the designed amplification pathway proceeded as expected (Figure 5B). The bands of the single strands and the A-B-C complex showed that the initial EDC precursor was properly assembled. When I1 was added, the band pattern of A-B-C changed and a new band appeared, suggesting that I1 was able to open the complex and start the strand-displacement reaction. After fuel strand F was introduced, further changes in mobility were observed, which agreed with recycling of I1 and release of DNAzyme strand B. We then checked whether the downstream substrate cleavage was caused by B specifically. The mixture of F and hairpin substrate S showed no obvious cleavage band, whereas the B+ S reaction produced the expected cleavage product, indicating that F did not nonspecifically cut or disrupt the substrate. The fluorescence spectra in Figure 5C gave a consistent result. Reporter alone, S + reporter, and B+ reporter all remained close to the background level, while the B+ S + reporter system produced a clear fluorescence signal. These observations show that the fluorescence output is generated only after DNAzyme-mediated cleavage of S and subsequent activation of the reporter.

**FIGURE 6:**
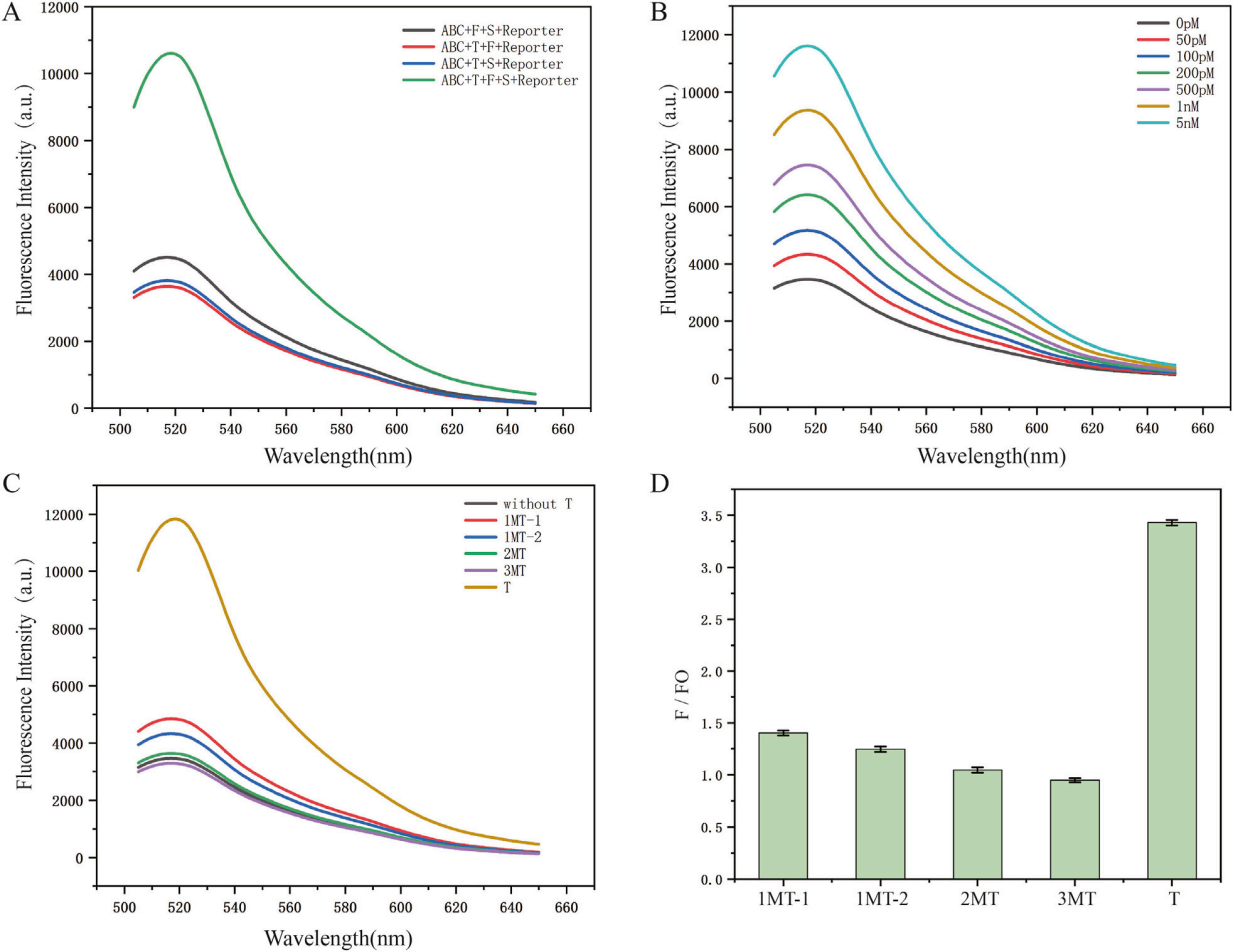
Analytical performance evaluation of the signal amplification biosensor. **(A)** Fluorescence emission spectra of the intact cascade system and the component-dropout control groups: (1) A-B-C + F + S + Reporter system (lacking target I1); (2) A-B-C + I1 + F + Reporter system (lacking substrate S); (3) A-B-C + S + I1 + Reporter system (lacking fuel strand F); (4) A-B-C + I1 + F + S + Reporter (intact cascade system). **(B)** Fluorescence emission spectra in response to varying concentrations of target I1 (0 pM–5 nM). **(C)** Fluorescence response spectra for the specific recognition of targets with different base mismatches. **(D)** Signal-to-noise ratio (SNR) analysis corresponding to the mismatched targets.

With the separate reaction steps verified, we next tested the complete EDC-DNAzyme sensing system and removed one component at a time to identify the source of the fluorescence signal (Figure 6A). The reaction without target I1 produced only a weak signal, suggesting limited spontaneous leakage from the A-B-C complex. The signal also remained close to the background when either fuel strand F or hairpin substrate S was omitted, showing that both target recycling and DNAzyme-mediated substrate cleavage were necessary for signal generation. By comparison, the full mixture containing A-B-C, I1, F, S, and the reporter gave a clear emission peak at approximately 520 nm. These results suggest that the fluorescence response arises from the coupled EDC reaction and downstream DNAzyme cleavage, rather than from nonspecific activation of the individual components. The sensitivity of the model sensing system was evaluated using target I1 at concentrations ranging from 0 pM to 5 nM. The fluorescence intensity increased with increasing I1 concentration, demonstrating a concentration-dependent response (Figure 6B). A distinguishable signal above the blank control was observed at 50 pM, indicating that the cascaded amplification design can effectively convert low-concentration nucleic acid inputs into measurable fluorescence outputs. We next assessed the sequence specificity of the biosensor using mismatched targets, including single-base mismatched sequences (1MT-1 and 1MT-2), a double-base mismatched sequence (2 MT), and a triple-base mismatched sequence (3 MT). Compared with the perfectly matched target, all mismatched sequences generated substantially lower fluorescence responses (Figure 6C). The corresponding signal-to-noise ratios further showed that nonspecific activation was strongly suppressed, particularly for the double- and triple-mismatched targets, whose signals approached the background level (Figure 6D). These results confirm that the biosensor maintains high sequence discrimination during amplified signal generation.

**FIGURE 7:**
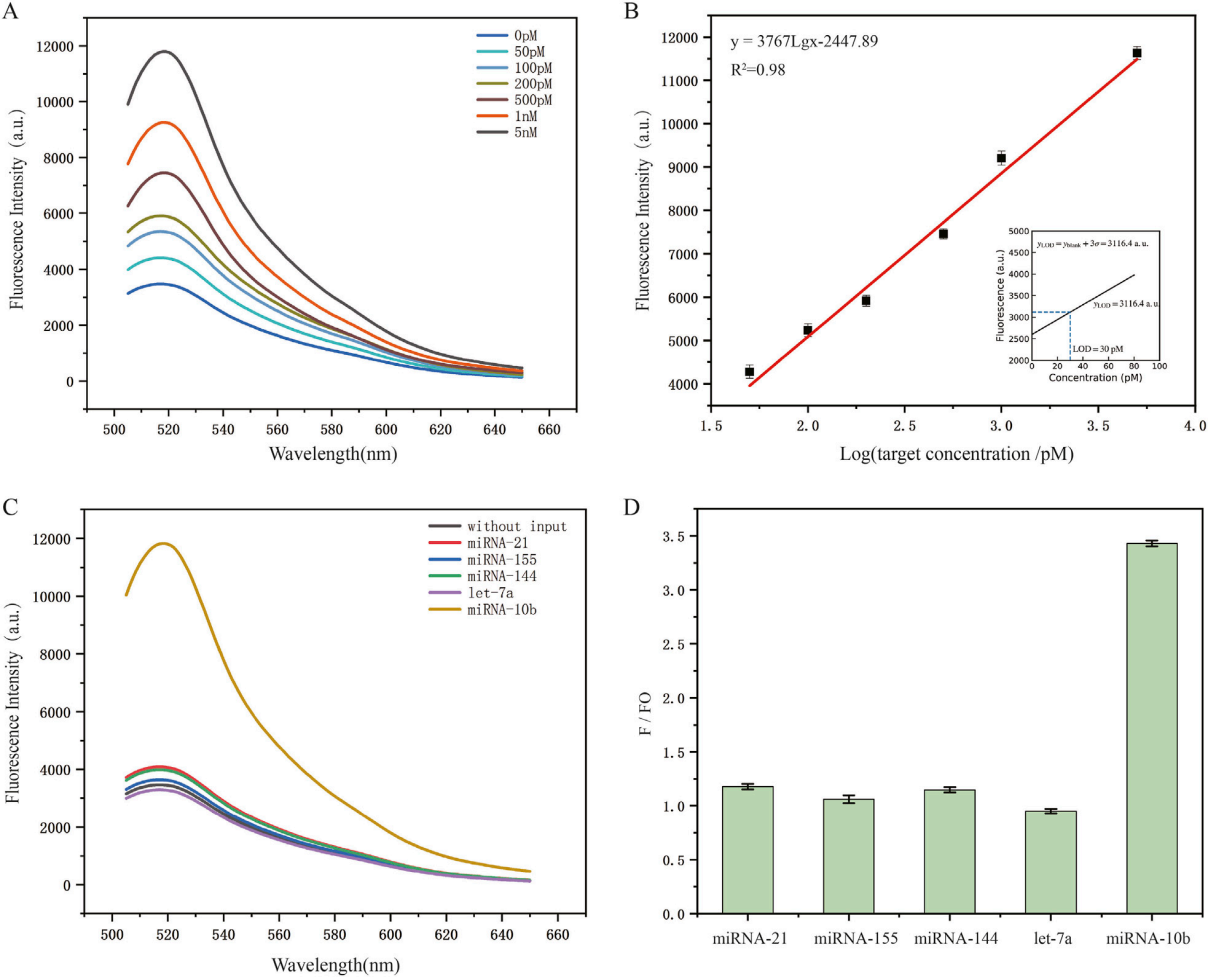
Analytical performance of the biosensor for miRNA-10 b detection. **(A)** Sensitivity analysis: Fluorescence emission spectra of the biosensor in response to varying concentrations of miRNA-10 b (ranging from 0 pM to 5 nM, from bottom to top). **(B)** Linear calibration curve between the absolute fluorescence intensity and the logarithm of the miRNA-10 b concentration. **(C)** Specificity analysis: Fluorescence response spectra of the system to the target miRNA-10 b and various non-target interfering miRNAs. **(D)** Bar chart of the signal-to-noise ratio (SNR) corresponding to different miRNA sequences.

We next tested the sensing module with miRNA-10b, a disease-related miRNA associated with tumor progression and metastasis. Because accurate detection of miRNA-10b requires both sensitivity and sequence selectivity, it was used here as the model target. The biosensor was incubated with miRNA-10b at concentrations from 0 pM to 5 nM at 37 °C for 300 min. As shown in Figure 7A, the fluorescence signal became stronger as the miRNA-10b concentration increased. When the fluorescence intensity was plotted against the logarithm of miRNA-10b concentration, a good linear response was obtained from 50 pM to 5 nM (Figure 7B). The fitted equation was y=3767⁡log⁡x−2447.89 , with $R^{2}=0.98$ . Based on the 3σ/k criterion and ten independent blank measurements, the calculated limit of detection was 30 pM. These results demonstrate that the EDC-DNAzyme cascaded biosensor enables sensitive detection of miRNA-10b under isothermal conditions without exogenous protein enzymes.

We further investigated the sequence selectivity of the biosensor toward miRNA-10 b by introducing non-target miRNAs, including let-7a, miRNA-144, and miRNA-155. As shown in Figures 7C,D, these non-target miRNAs produced fluorescence signals close to the background level, whereas miRNA-10 b induced a strong fluorescence response. This result indicates that the biosensor can distinguish the target miRNA from unrelated interfering miRNA sequences and initiate the amplification cascade in a target-dependent manner. Compared with enzyme-assisted amplification methods listed in Supplementary Table S1, this system avoids exogenous protein enzymes such as polymerases, nucleases, and CRISPR/Cas effectors. Although some enzyme-assisted assays may reach lower detection limits, the isothermal design, which does not require exogenous protein enzymes, improves compatibility with programmable DNA circuits and facilitates integration with the upstream DNAzyme-based computing module. Overall, the EDC-DNAzyme cascaded amplification module provides a sensitive and sequence-selective sensing front-end for linking trace miRNA recognition with molecular logic computation.

The specificity tests further showed that the biosensor responded preferentially to miRNA-10b rather than to the tested non-target miRNAs. The protected hairpin substrate and the cascaded EDC-DNAzyme reaction helped restrict nonspecific signal generation, so the interfering sequences produced only weak fluorescence signals. Even in the presence of closely related nucleic acid sequences, the system maintained a clear difference between the target and control groups. This target-dependent response suggests that the proposed design may help reduce false-positive signals in future miRNA detection applications.

## 4 Discussion

In this study, we built an allosterically regulated DNAzyme system that links molecular computation with nucleic acid sensing that does not require exogenous protein enzymes. In this design, DNAzyme activity is not limited to producing a final fluorescence readout. Instead, it is controlled at different reaction steps to participate in gate operation, signal amplification, and stepwise signal transfer. The computing part relies on allosteric switching of DNAzyme activity, while the sensing part uses entropy-driven catalysis to strengthen weak nucleic acid inputs before they enter the logic network. In this way, low-level target signals can be converted into amplified DNA signals and then processed by the downstream DNAzyme-based computing modules.

For molecular computation, we constructed three basic DNAzyme logic units, including co-activated AND logic, threshold, and subtraction gates. These modules correspond to key operations required for reliable clinical diagnostics: strict coincidence detection, binary background suppression, and differential signal processing. The successful cascade of these gates demonstrates that allosteric DNAzyme regulation can support robust integrated signal analysis tailored for practical applications. In particular, the integrated cascaded architecture shows that DNAzyme-based systems can transmit signals across multiple layers while maintaining directional signal propagation through irreversible substrate cleavage. This design provides a useful strategy for building more sophisticated nucleic acid computing circuits with reduced signal retroactivity and improved modularity.

For biosensing, we developed an EDC-DNAzyme cascaded amplification module to address the problem that trace miRNA targets are often insufficient to directly activate downstream molecular circuits. In this module, the target strand first initiates the EDC reaction to release an active DNAzyme, and the released DNAzyme subsequently cleaves a hairpin substrate to generate both a fluorescence output and a secondary trigger for self-feedback amplification. This dual-cycling design combines target recycling with DNAzyme-mediated signal regeneration, allowing the system to amplify weak nucleic acid inputs under isothermal conditions without exogenous protein enzymes. The biosensor detected miRNA-10 b over a linear range from 50 pM to 5 nM and achieved a limit of detection of 30 pM. It also showed strong sequence discrimination against mismatched targets and non-target miRNAs, indicating that the allosterically protected cascade design can suppress nonspecific activation while preserving target-dependent amplification.

Compared with enzyme-assisted amplification systems (Supplementary Table S1), the present platform avoids the use of exogenous protein enzymes such as polymerases, nucleases, or CRISPR/Cas effectors. This design, which does not require exogenous protein enzymes, may improve compatibility with programmable DNA circuits and reduce the complexity associated with enzyme storage, reaction optimization, and multi-enzyme coupling. Although some enzyme-assisted assays can achieve lower detection limits, their direct integration into multilayer molecular computing networks remains challenging. In contrast, the EDC-DNAzyme module developed here generates nucleic acid outputs that are intrinsically compatible with downstream strand-displacement and DNAzyme logic circuits, making it suitable as a sensing front-end for molecular computation.

Nevertheless, several limitations should be noted. First, the current biosensing experiments were mainly performed under controlled buffer conditions. Although the achieved sensitivity and specificity suggest potential analytical utility, further validation in complex biological samples, such as human serum or plasma, will be required before practical diagnostic application. Second, although the current system demonstrates a cascaded logic network, larger-scale networks involving multiple biomarkers and multiple simultaneous target inputs remain to be developed. Future work will focus on improving multiplexing capability, accelerating reaction kinetics, and integrating the sensing front-end with more robust molecular logic circuits for future diagnostic applications.

## 5 Conclusion

In summary, this work presents an allosteric DNAzyme-based modular system that integrates signal amplification without exogenous protein enzymes with programmable molecular logic analysis. By constructing co-activated AND logic, thresholding, and subtraction gates, we demonstrated a DNAzyme-based integrated logic circuit capable of hierarchical signal processing. By coupling EDC with DNAzyme-mediated self-feedback amplification, we further established a sensitive and sequence-selective biosensor for miRNA-10 b detection, achieving a detection range of 50 pM to 5 nM and a detection limit of 30 pM. This integrated strategy provides a proof-of-concept nucleic acid platform for linking trace biomarker recognition with molecular logic computation, offering a potential foundation for future intelligent molecular diagnostic systems.

## Data Availability

The datasets presented in this study can be found in online repositories. The names of the repository/repositories and accession number(s) can be found in the article/Supplementary Material.
