# Supplementary material for: An allosteric DNAzyme-Based modular system for dynamic computation and sensitive MicroRNA detection
Source: Front Bioeng Biotechnol. 2026 Jul 29;14:1895332. doi: 10.3389/fbioe.2026.1895332 (PMC13461625; doi:10.3389/fbioe.2026.1895332)
Supplement: Supplementary file 1 [file Table1.docx]

**SUPPLEMENTARY TABLE**

Table 1. Comparison of the proposed biosensor with other miRNA detection methods.

| Detection methods | Linear range | LOD | Reference |
| --- | --- | --- | --- |
| Fluorescence | 0.01–3nM | 1.14pM | (Lin et al., 2025) |
| Fluorescence | 0.3–15pM | 0.15pM | (Zang et al., 2025) |
| Colorimetry | 8.3–74nM | 3.7pM | (Zhang et al., 2025) |
| Fluorescence | 0.4–53.6nM | 0.25nM | (Lan et al., 2025) |
| Fluorescence | 0.2–6nM | 22.9pM | (Pan et al., 2020) |
| Colorimetry | 25 pM–5nM | 21.7pM | (Pritchard et al., 2012) |
| Fluorescence | 25 pM–6nM | 9.71pM | (Liang et al., 2026) |
| Fluorescence | 49 nM–3135nM | 100pM | (Lu et al., 2025) |
| Fluorescence | 50pM–10nM | 30pM | This work |

Table 2. Sequences of miRNAs used for specificity assay.

| Name | Sequence (5′ − 3′) |
| --- | --- |
| miRNA-10b | UACCCUGUAGAACCGAAUUUGUG |
| let-7a | UGAGGUAGUAGGUUGUAUAGUU |
| miRNA-144 | GGAUAUCAUCAUAUACUGUAAGU |
| miRNA-155 | UUAAUGCUAAUUGUGAUAGGGGU |
| miRNA-21 | UAGCUUAUCAGACUGAUGUUGA |

Table 3. Sequences of the oligonucleotides used in this study.

| Name | Sequence(5'-3') |
| --- | --- |
| Z1 | TAGTGTATGTTCAGCGATACGGTAGAAGAATGTGTTATCTTCTACCGTCACCCATGTTAACTCTT |
| X1* | CATTCTTCTACCGTATCGCTTAGCTAA |
| W1* | TTCTGTTCATGGGTGACGGTAGA |
| R1 | ROX-TTGCATGTTGACGAGCTCACCCATGAAGAGTTAT/rA/GGAACATACACTA-BHQ2 |
| W1 | TCTACCGTCACCCATGAACAGAA |
| X1 | TTAGCTAAGCGATACGGTAGAAGAA |
| Z2 | TAGTGTATGTTCAGCGATAGCTCGTCTTGTTGACGAGCTCACCCATGTTAACTCTC |
| T1* | CATGGGTGAGCTCGTCAACATGCAACA |
| R2 | TET-TTGTTGTATAGTACCGTCACCACAGAGAGTTAT/rA/GGAACATACACTA-BHQ1 |
| Xuax1 | CTCTTCTCTATTCATGGGTGGACTCTGCAA |
| T1(O1) | TTGCATGTTGACGAGCTCACCCATGAAGAGT |
| Z3 | TAGTGTATGTTCAGCGATACGGTACTTGTATAGTACCGTCACCCATGTCTCTTCTC |
| T2* | TATAGTCGGGTGACGGTACTATACAAGTAC |
| R3 | 6-FAM-GAGAAGAGT/rA/GGAACATACACTA-BHQ1 |
| Anh1 | TCGGGTGACGGTACTATACAACAACATATCAATTCT |
| Anh2 | TGTGAGATGAATTGATATGTTGTTGTATAGTAATTC |
| T2(O2) | TTGTTGTATAGTACCGTCACCCGACTATCGGCAT |
| S1 | AACAACATATCAATTCATCTCACACAACAACCA |
| I1 | TACCCTGTAGAACCGAATTTGTG |
| A | CTCATTACCCTGTAGAACCGA |
| B | CGCATCTTTCCGAGCCGGTCGAAAACGAATTTGTG |
| C | CACAAATTCGGTTCTACAGGGTAATGAGCACAAATTCGTTTTTAAGATGCG |
| F | ATCTTAAAAACGAATTTGTGCTCATTACCCTGTAGAACCGA |
| S | CAAACACAATCACACACAAATTCGTT/rA/GAAGATGCGTACCCTGTAGAACCGAATTTGTG |
| I2 | AGAAGATGCGTACCCTGTAGAACCGAATTTGTG |
| Output | CAAACACAATCACACACAAATTCGTT |
| Reporter | BHQ1-CAAACACAATCACACACATTAAAACGAATTTGTGTGTGATTGTGTTTG-6-FAM |

**2. SUPPLEMENTARY NOTES1. EXPLANATION OF FLUOROPHORE–QUENCHER DISTANCE IN R1/R2**

Although the fluorophore and quencher may appear separated along the primary sequences of R1 and R2, the effective quenching efficiency is determined by the spatial conformation of the assembled DNAzyme–substrate complexes rather than by linear sequence length alone. In the assembled state, substrate hybridization with the DNAzyme binding arms, local folding, and conformational constraints can reduce the effective fluorophore–quencher distance and maintain a relatively low background signal before cleavage. This is consistent with the low fluorescence background observed in the no-input and single-input control reactions

**REFERENCES**

Lan, L., Yang, Z., and Lan, M. (2025). Near-infrared light-activated dna biosensor for sensitive detection of mirnas via a catalytic hairpin assembly signal amplification strategy. ACS omega 10, 54691–54699

Liang, L., Xu, B., Xiao, S., Mu, X., Zhao, S., and Tian, J. (2026). A new split dna-based activation of crispr/cas12a for amplification-free and dual-stimulus responsive detection and precise imaging of mirna-221. Talanta , 129443

Lin, X., Yang, Y., Zhu, W., He, X., and Liu, Y. (2025). Near-infrared dna biosensors based on polysulfonate coatings for the sensitive detection of micrornas. Nanoscale Advances 7, 549–559

Lu, Y., Xu, J., Liu, S., Tian, B., and Long, F. (2025). A facile optical fiber-embedded microfluidic biochip for rapid and sensitive detection of microrna-let-7a in serum. Microchimica Acta 192, 9

Pan, L., Zhang, H., Zhao, J., Li, X., Xu, R., Mo, Y., et al. (2020). A highly sensitive and selective signal-on strategy for microrna quantification. Analytica chimica acta 1100, 258–266

Pritchard, C. C., Cheng, H. H., and Tewari, M. (2012). Microrna profiling: approaches and considerations. Nature Reviews Genetics 13, 358–369

Zang, J., Wang, T., Zhang, F., Zhang, M., Chen, M., Liu, K., et al. (2025). Mitochondrial microrna detection using a sequentially activatable allosteric dna biosensor for in vivo molecular visualization of tumors and tumor drug resistance. Biosensors and Bioelectronics , 118046

Zhang, J.-N., Liu, F.-M., Du, X.-J., and Zhao, X.-L. (2025). Dual-mode, signal-amplified dna biosensor for label-free, reliable assay of gestational diabetes mellitus-related mirna (mir-135a). Journal of Pharmaceutical and Biomedical Analysis 253, 116565
